# Effect of isokinetic eccentric training on the human shoulder strength, flexibility, and muscle architecture in physically active men: A preliminary study

**DOI:** 10.1371/journal.pone.0293439

**Published:** 2023-12-19

**Authors:** Sebastian Vetter, Pierre Hepp, Axel Schleichardt, Stefan Schleifenbaum, Maren Witt, Christian Roth, Hans-Peter Köhler

**Affiliations:** 1 Department of Biomechanics in Sports, Leipzig University, Leipzig, Germany; 2 Department for Orthopedics, Trauma and Plastic Surgery, Leipzig University, Leipzig, Germany; 3 Department of Biomechanics, Institute for Applied Training Science, Leipzig Germany; 4 Department of Pediatric Radiology, Leipzig University, Leipzig, Germany; Erzurum Technical University: Erzurum Teknik Universitesi, TURKEY

## Abstract

Strengthening the rotator cuff muscles is important for injury prevention and rehabilitation. Since muscle fascicle length improves motor performance and is suggested to reduce the risk of injury for the hamstring, it may be an important variable to promote multidirectional changes in the function and macroscopic structure for the shoulder. Recent literature reviews overwhelmingly suggest that eccentric exercises improve fascicle length and functional measures for the lower limb. However, there is a research gap for the shoulder. Since ultrasound imaging is the most commonly used imaging technique to quantify muscle structure, but has yielded heterogeneous results in different studies, there is another issue and a research gap for the imaging method. Based on the research gaps, the purpose of this study was to evaluate the effects of standardized eccentric strength training on the function and structure of the external rotator cuff muscles using an isokinetic dynamometer and MRI. Therefore, a preliminary pre-post intervention study was conducted and 16 physically active men were recruited in October 2021. For the right shoulder, an eccentric isokinetic training was performed twice a week for almost six weeks. The primary outcome measures (external rotators) were active and passive range of motion, eccentric and concentric torque at 30, 60, and 180°/s isokinetic speed, and fascicle length and fascicle volume for the supraspinatus and infraspinatus muscles. The findings show a training effect for the absolute mean values of eccentric strength (+24%, *p* = .008). The torque-angle relationship increased, especially in the final phase of range of motion, although a 4% (*p* = .002) decrease in passive range of motion was found in the stretch test. Positive changes in muscle structure were shown for the supraspinatus muscle fascicle length (+16%, *p* = .003) and fascicle volume (+19%, *p* = .002). Based on the study results, we can conclude that eccentric isokinetic training has a significant positive effect on the shoulder. To our knowledge, this is the first eccentric training study using both isokinetic dynamometer and muscle diffusion tensor imaging to access functional and structural changes in the human shoulder rotator cuff muscles. The methods were shown to be applicable for interventional studies. Based on these results, populations such as high-performance handball players with highly trained shoulders should be included in future studies.

## Introduction

Shoulder injuries are not only an ongoing problem in general practice, they are also widespread, with an average incidence of 29.3 per 1000 person-years, making the shoulder the third most common site of complaints in the UK and the Netherlands [1]. Moreover, because shoulder complaints can lead to injury and prolonged rehabilitation [1], the lifetime prevalence of shoulder problems is approximately 31% [2]. Despite these high incidence rates, because the shoulder is a muscle-driven joint and therefore highly modifiable, there may be great potential to prevent shoulder injuries by addressing injury risk factors such as joint range of motion (ROM) and strength [3–7]. Another promising key factor to improve these risk factors is the muscle fascicle length (FL). A longer muscle fascicle improves motor performance [8–10], and has been suggested as risk factor for the hamstrings [11]. Although the injury mechanisms for the shoulder are not well understood [12–14], an increased FL may act as a buffer to reduce the high internal rotation eccentric stretching stress found after a throwing performance (deceleration moment) [15], which partially explain the high incidence of supraspinatus tendon lesions and impingement syndromes [12, 14]. Therefore, a recent meta-analysis [16] suggests, that future studies should focus on the effects of resistance training modalities on the upper extremity muscle fascicle geometry due to its strong relationship with athletic performance, injury, and force production. However, it seems that the majority of exercise interventions aimed at reducing the risk of shoulder injury and discomfort target functional changes in flexibility and strength without substantially reducing the injury rate [17–19].

In contrast, a so-called eccentric training seems to fill the gap due to its multidirectional effects on function and muscle structure [20–22]. Eccentric-only training is characterized by high-intensity resistance training during the decelerating movement, while the muscle is lengthened under active muscle contraction. This effectively induces tissue damage and a hypertrophic response [23, 24]. Therefore, eccentric training has been proposed as an effective intervention strategy to reduce strain injuries [25–27], which can be achieved by increasing muscle FL [20, 22].

Besides a systematic review on the effects of general resistance training on muscle parameters for the upper extremity [16], there is a lack of eccentric training studies focusing on the musculature of the shoulder joint [28]. Another issue facing eccentric training studies is the standardization of the training stimulus, the ROM testing, as well as for the imaging method to quantify muscle structural changes. For the shoulder joint, there are only a few studies that include eccentric training without high intensity exercises and for rehabilitation purposes [29–34]. Since it was introduced that diagnosis of ROM is another issue due to the widespread manual measurement [29, 30], there is a research gap for studies using an isokinetic device to standardize training and testing for flexibility measures. Another issue is that structural changes are commonly obtained using 2D ultrasound imaging [16], but even less is known about the three-dimensional changes in muscle fiber architecture. Furthermore, Yagiz and colleagues [20] showed that ultrasound assessment and extrapolation technique lead to problems in interpretation due to heterogenous results for FL changes. To solve this problem, as a promising approach MRI-based muscle diffusion tensor imaging (mDTI) [35–37] seems worth to consider for further studies to inspect better three-dimensional muscle structural changes and raise standardization in acquisition.

Based on the issues in imaging techniques and the research gaps for standardized eccentric training and testing for the shoulder, the aim of this study was to investigate structural and functional changes following a six-week isokinetic eccentric strength training program for the dominant shoulder in a healthy and physically active male population. In addition, this study will test the feasibility of a newly developed isokinetic functional stretch test and apply mDTI to the shoulder. Main outcome measures (external rotators) were active ROM (aROMmax), submaximal and maximal passive ROM (pROMsub and pROMmax), maximal eccentric and concentric isokinetic strength, and analysis of the torque-angle relationship. To quantify changes in the infraspinatus and supraspinatus muscles, mean fractional anisotropy (FA), FL, and fascicle volume (FV) were calculated based on mDTI.

## Materials and methods

This is a preliminary experimental study [38]. Volunteers were recruited in October 2021. The study protocol was approved by the Leipzig University ethics committee (ethical approval nr: 362/21-ek) and is registered in the German Clinical Trials Register (DRKS00032375). The study was performed according to the declaration of Helsinki. All subjects gave written informed consent. The authors had no access to information that could identify individual participants during or after data collection.

### Sample

The sample consisted of 16 active male student athletes for isokinetic measurements (23.3 ± 3.9 years; 181.2 ± 6.9 cm body height; 76.4 ± 7.4 kg body mass). The sample size was based on studies that calculated or suggested at least 16 subjects [22, 39] to achieve an effect size >1.00 with 80% power and an alpha error of 0.05% reported for the lower limb, and another study for the shoulder that included 14 subjects [34].The physical activity of the recruited population is characterized by strength training and individually varied activities such as skiing, judo, soccer, running, handball, or javelin throwing at least twice a week. Out of this population eleven subjects were randomly chosen for magnetic resonance imaging (MRI). All participants were required to have a healthy dominant right shoulder. Subjects were excluded, if they showed a history of musculoskeletal disorders, had regular medication, discomfort, pain, or known lesions in the right shoulder. In addition, five days prior to data collection, participants were instructed to discontinue any intense and exceptional physical activity or heavy training.

### Eccentric isokinetic resistance training intervention

A total of twelve eccentric isokinetic strength training sessions for the external rotator cuff muscles were performed over a period of six weeks (twice a week). Training was performed using BTE Primus RS isokinetic dynamometer (Baltimore Therapeutic Equipment Company, Hanover, MD, USA). Prior to the start of the intervention, all subjects completed two sessions of familiarization with the eccentric training and testing procedure.

The training protocol followed the recommendations from Toigo and Boutellier [40] and was standardized in terms of weeks and days of training, ROM, movement velocity, load magnitude, number of sets and repetitions, and exercise execution regulations (Table 1). After two weeks of intervention, the total ROM was shifted 15 degrees toward the internal ROM and the load level was increased based on the load level of the last three sessions. The training was performed in prone position with 90° shoulder abduction and 90° elbow flexion.

**Table 1:** Eccentric resistance exercise determinants.

Resistance exercise determinants	Eccentric isokinetic internal rotation
Number of repetitions	10 repetition
Load magnitude	± 10% of 10 Repetition Maximum
Time under tension	~4 seconds
Rest in-between repetitions	~4 seconds
Volitional muscular failure	no
Range of motion	120° (from 60° external to 60° internal rotation)
Movement speed	30°/s
Exercise execution	Prone Position
	Fixed joints: elbow (90° flexion) and Hand (0° neutral)
	rotating joint: shoulder (90° abduction)
Fractional and temporal distribution of the contraction modes per repetition and duration of a repetition	4 seconds eccentric
	0 seconds isometric
	0 seconds concentric
Number of sets	5 Sets
Rest in between sets	120 seconds
Number of exercise interventions	2/ week
Wamp-Up	Shoulder rotation and pull-ups
Recovery time in-between exercise sessions	48–76 hours
Duration of experimental period	5.5 weeks

### Data acquisition

**Fig 1:**
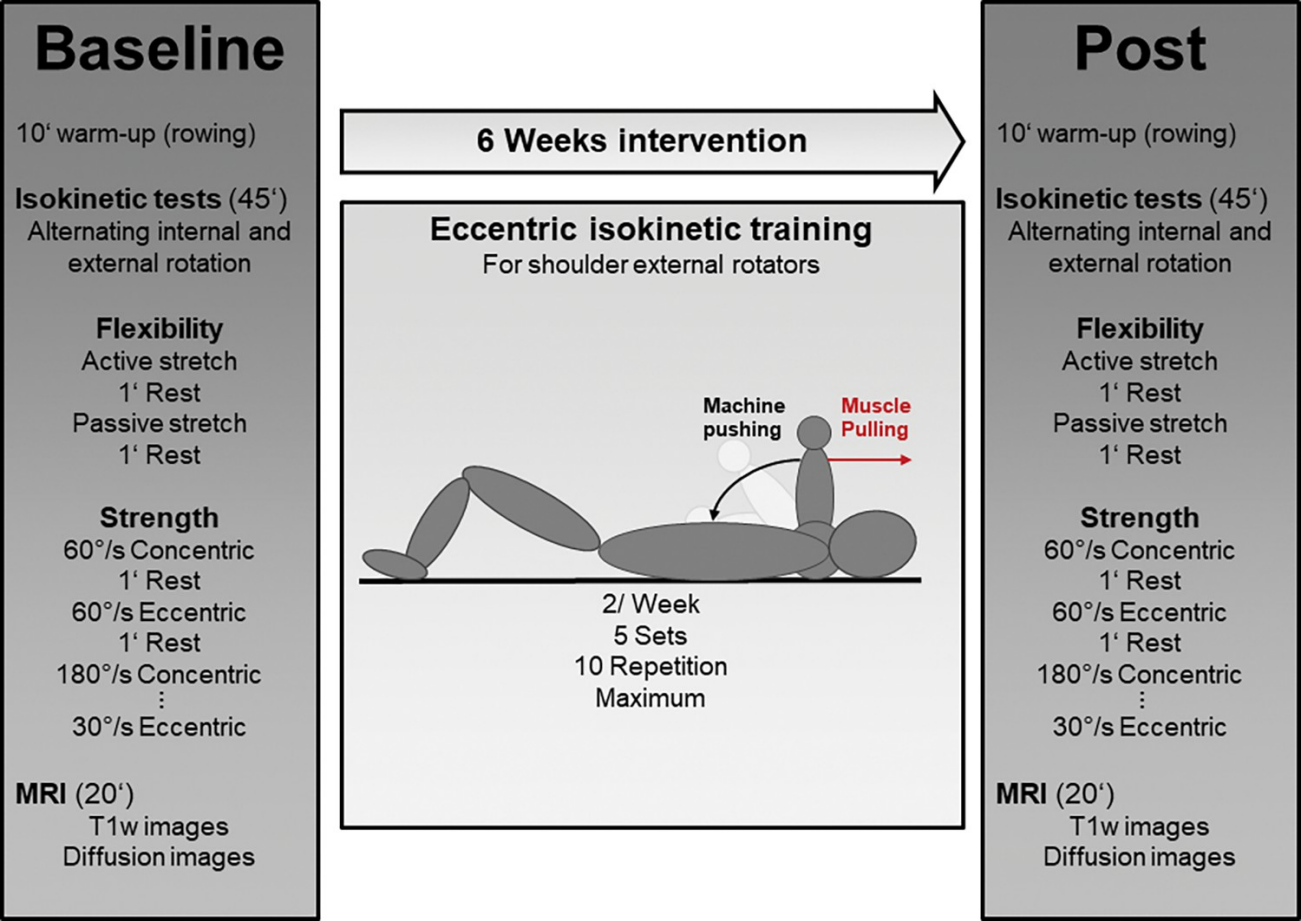
Experimental design.

Functional and structural parameters of the right shoulder were assessed using an isokinetic dynamometer and MRI device three to five days before the first (pre) and after (post) the six week eccentric intervention period. At pre- and posttest, the subjects began with the isokinetic functional diagnostics for the internal and external rotator cuff muscles prior to MRI. First, the active stretch test was performed, followed by the passive stretch test. Second, the maximal isokinetic strength tests were performed separately for concentric and eccentric and in the order of 60°/s, 180°/s and finally at 30°/s movement velocity. Each test was separated by 60 seconds. Then, functional isokinetic diagnostics were followed by MRI, which included two sequences for the right shoulder as explained below (Fig 1).

### Isokinetic testing

**Fig 2:**
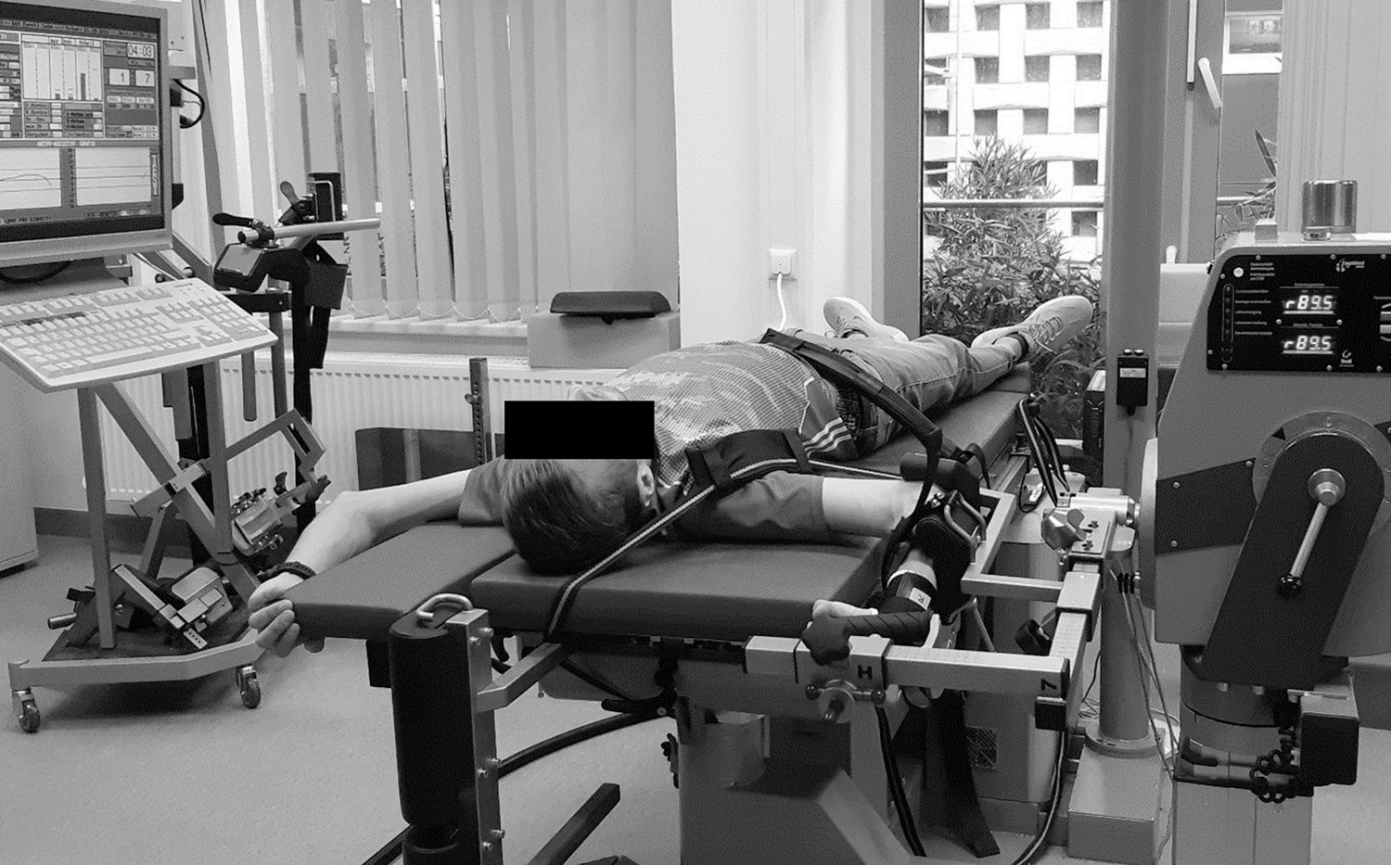
Functional testing using IsoMed 2000 isokinetic dynamometer.

IsoMed 2000 (D&R Ferstl GmbH, Hemau, Germany) was used to test stretch behaviour and strength of the right shoulders external and internal rotator cuff muscles. The isokinetic diagnostics and testing setup is inspired by recommondations [41] and is similar to other studies [42, 43]. Data were recorded at 200 Hz. Each subject was tested in the supine position with fixed shoulder-arm joints (Fig 2) as for the eccentric training described (Table 1). In addition, the shoulder was fixed ventrally to prevent shoulder elevation in an individually standardized manner. Subjects were tested in the order in which the tests are described.

Active and passive stretch tests consisted of two familiarization trials and five test trials alternating between internal and external rotation. The participants eyes were closed during both tests. Volunteers were instructed to move the apparatus slowly and with ease until aROMmax. The turning point were reached when the subject was unable to continue the antagonist stretch generated by the agonist muscles. The following passive stretch test was performed at an isokinetic speed of 10°/s. The passive stretch was performed until pROMmax was reached.

The following voluntary maximum strength tests were performed separately for concentric and eccentric action, each dynamically and alternating between internal and external rotation movement. Strength measurements were performed over a 160° ROM (70° internal rotation and 90° external rotation). Each subject’s position and dynamometer adapter gravity correction value and settings were individualized at pretest and used for posttests (Fig 2).

### Magnetic resonance imaging

A 3-Tesla Siemens MAGNETOM Prisma scanner (Siemens Healthcare, Erlangen, Germany) with a shoulder coil (XL, 16-channel) was used for MRI scans. The participants lay in a head first supine position with the right arm in the neutral position and the hand supinated. The MR protocol consisted of a T1-weighted and a diffusion-weighted sequence. The T1-weighted sequence consisted the following parameter settings: repetition and echo time TR/TE = 492/20 ms, slice thickness = 0.7 mm, flip angle = 120°, field of view = 180 x 180 mm^2^, matrix = 256x256 mm^2^. Diffusion-weighted sequence were acquired as following: repetition and echo time TR/TE = 6100/69 ms, slice thickness = 5.2 mm, flip angle = 90°, field of view = 240 x 240 mm^2^, matrix = 122 x 122 mm^2^, 48 diffusion sampling directions with b = 400 s/mm^2^. Total scan time reached 12 minutes.

### Data processing

MATLAB v.R2022a (MathWorks, Natick, USA) was used to process the isokinetic dynamometer data. Stretching parameter calculations were based on the average of five trials in each test. pROMmax was calculated when subjects reached the end point characterized by 8 Nm. For further analysis of changes in the morphology of the passive torque-angle curves, a three-parametric e-function was fitted from each subject’s individual offset start angle to the 100° internal rotation and 130° external rotation end points (100% stretch), which are shown in percent stretch in the figures. The e-function was then used to calculate the angle at the instant of 0.01 Nm/degree (pROMsub) and to calculate the fit to the defined end points using a statistical parametric mapping (SPM1d) method in MATLAB. SPM is a vector field analysis method which was found to be more appropriate in evaluating differences across a whole time-series of data points which also occurs for isokinetic dynamometry resulting in moment-angle curves [44]. This complex flexibility analysis methodology was evaluated and developed in-house and is described in the S1 Appendix.

For strength analysis, the mean of three maximum consecutive repetitions were used. Raw data were filtered using a 6 Hz cutoff frequency [45]. From each test, the acceleration and deceleration phases were cut, leaving only the interval with the desired isokinetic velocity. The maximum torque was then identified in the isokinetic phase and the mean torque was calculated over this phase. For inferential statistics and comparison of curve morphology using SPM1d, data were normalized to body mass [45, 46].

**Fig 3:**
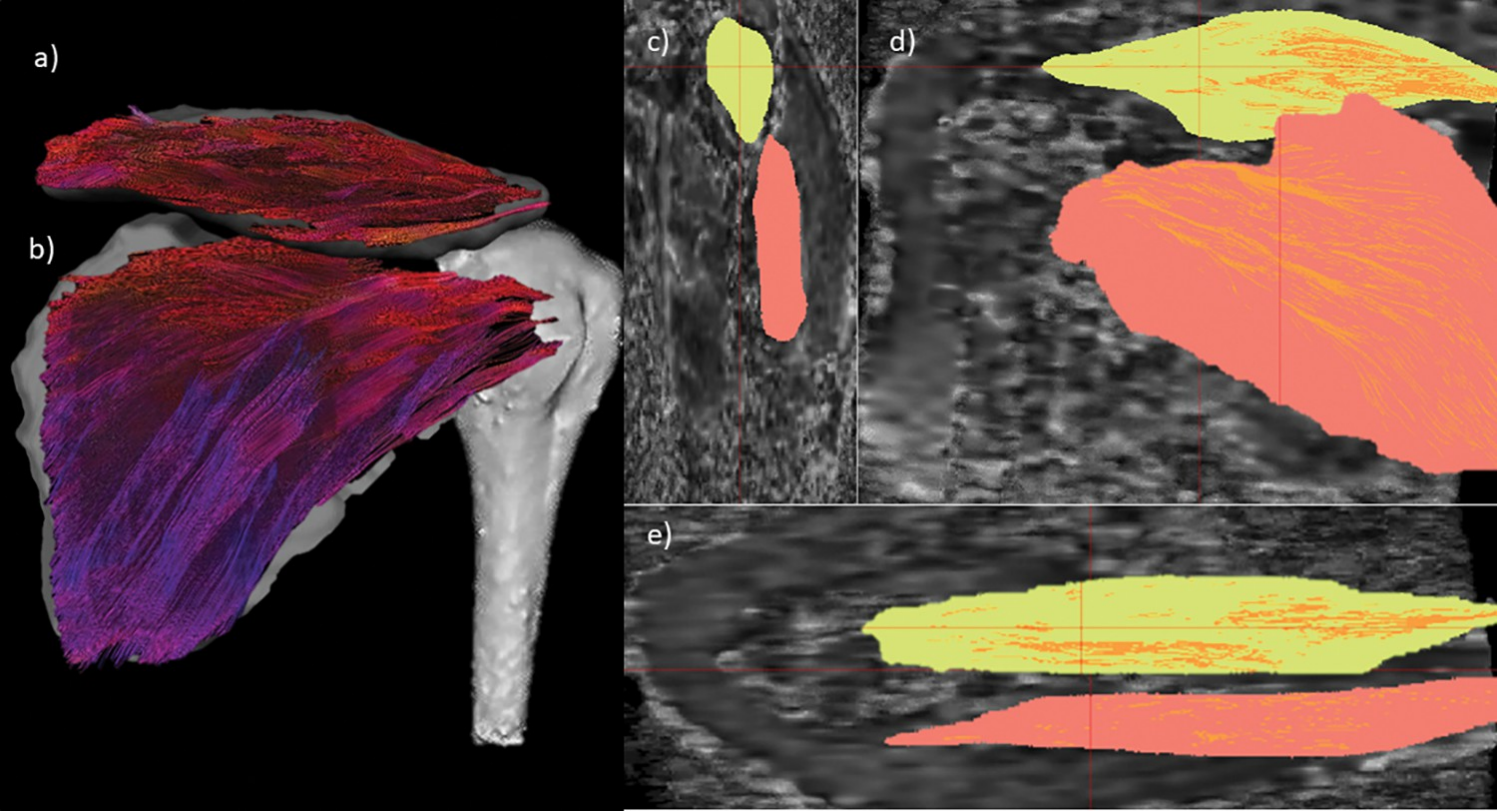
Right shoulder dorsal view tractography. a) coronal plane supraspinatus muscle fiber with directional coloring, b) infraspinatus muscle, c-e) sagittal, coronal and axial view on diffusion-weighted-images and volume of interest.

MRI data were first processed using Mimics Materialise v.24.0 (Leuven, Belgium) for manually segment the supraspinatus and infraspinatus muscles to extract a volume of interest (VOI). Then, the diffusion-weighted images were processed and corrected using DSI Studio (v. 3rd of December 2021, http://dsi-studio.labsolver.org). Based on VOI-based deterministic fiber tractography, FA, muscle FL and FV were calculated using the integrated statistics tool in DSI Studio (Fig 3). This method was evaluated in a previous study and showed excellent reliability for the tractography outcome measures [47].

### Statistics

MATLAB v.R2022a (MathWorks, Natick, USA) and SPSS v.27 (IBM, Armonk, New York, USA) were used for statistics. Descriptive results were based on mean and standard deviation (±). Participants were excluded from further analysis if the data distribution showed outliers and z-transformed values exceeded 2.5. Repeated measures multivariate analysis of variance (MANOVA) was used to show main effects and interactions between different factors. For strength statistics, values are normalized to body mass and the factors were time (pre and post), mode (eccentric and concentric), and speed (30°/s, 60°/s and 180°/s). For flexibility parameters, the factors were time (pre and post) and test (aROM and pROM). For muscle parameters, the factor for statistical analysis were time (pre and post). Statistics were separated for the muscles.

For further post-hoc mean comparisons, paired t-tests were used. The *p*-value was set at 0.05. Differences between the torque-angle curves were calculated using SPM1d integrated in MATLAB.

## Results

All 16 subjects completed twelve training sessions within six weeks. The data were tested for normal distribution and homogeneity. One outlier was excluded for analysis of the supraspinatus muscle, three subjects were excluded due to invalid test performance for the active stretch test and four subjects were excluded for analysis of the infraspinatus muscle due to a truncated proximal field of view on MRI.

### External rotator cuff muscles functional and structural changes

A three-factors repeated measures MANOVA were calculated for external and internal rotator cuff muscles strength. Overall, the external rotator cuff muscles showed a significant time x mode interaction for the parameter mean torque (F(1,14) = 5.82; *p* = .030; ηp^2^ = .293) and peak torque (F(1,14) = 3.58; *p* = .080; ηp^2^ = .203). Also, an interaction effect was found for time x speed for mean torque (F(1,14) = 5.29; *p* = .011; ηp^2^ = .274) and peak torque (F(1.429,20.004) = 3.51; *p* = .063; ηp^2^ = .200). The interaction effect of time x mode was significant for the 30°/s velocity strength tests (Wilks-Lambda = .450; F(2,13) = 7.93; *p* =. 006; ηp^2^ = .550).

For analysis of the stretching tests and ROM results, a one-way MANOVA showed significant pre-post decreases for the trained external rotator cuff muscle parameters (Wilks-Lambda = .572; F(2,14) = 5.25; *p* = .020; ηp^2^ = .428). Furthermore, the analysis for the aROMmax revealed a significant pre-post decrease for the internal rotator cuff muscles (t(12) = 2.37; *p* = .018; d = .657). The pre-post differences for the external rotator cuff muscles for each factor and parameter can be found in Table 2.

**Table 2:** Post-hoc comparisons for the external rotator cuff muscles.

Muscle	parameter		Pre	Post	Δ%	*p*	*d*
External rotator cuff muscles	Strength eccentric (Nm)						
	Mean torque	30°/s	28.68 (6.31)	35.57 (6.58)	+24.02	.008	0.676
		60°/s	33.01 (6.92)	37.42 (7.31)	+13.36	.098	0.339
		180°/s	36.60 (7.97)	40.73 (8.42)	+11.30	.145	0.274
	Peak torque	30°/s	37.70 (7.76)	44.04 (7.80)	+16.82	.031	0.503
		60°/s	41.12 (8.25)	45.34 (8.93)	+10.25	.150	0.268
		180°/s	42.50 (8.94)	46.22 (9.29)	+8.76	.190	0.227
	Strength concentric (Nm)						
	Mean torque	30°/s	22.55 (4.52)	23.07 (4.10)	+2.32	.451	0.071
		60°/s	22.86 (4.23)	24.45 (4.57)	+6.93	.227	0.193
		180°/s	21.75 (4.55)	21.48 (4.14)	-1.29	.374	0.082
	Peak torque	30°/s	28.61 (5.54)	30.49 (5.41)	+6.57	.226	0.193
		60°/s	29.41 (5.25)	31.38 (6.31)	+6.70	.253	0.171
		180°/s	25.69 (4.63)	25.23 (4.77)	-1.81	.345	0.101
	Flexibility (internal rotation)						
	Active stretch	aROMmax (°)	71.59 (8.62)	66.82 (7.72)	-6.66	.018	0.657
	Passive stretch	pROMsub (°)	60.95 (10.70)	58.06 (9.48)	-4.74	.009	0.667
		pROMmax (°)	86.63 (9.65)	83.46 (8.50)	-3.66	.002	0.824
Infraspinatus	Fascicle length (mm)		66.55 (6.15)	64.63 (4.80)	-2.89	.277	0.237
	Fascicle volume (mm^3^)		257.83 (30.21)	258.29 (35.33)	+0.18	.492	0.008
	Fractional anisotropy		0.2500 (0.02)	0.2486 (0.02)	-0.56	.690	0.158
Supraspinatus	Fascicle length (mm)		36.76 (6.93)	42.68 (7.94)	+16.1	.003	1.117
	Fascicle volume (mm^3^)		143.17 (29.34)	170.54 (23.42)	+19.12	.002	1.207
	Fractional anisotropy		0.2855 (0.02)	0.2845 (0.02)	-0.35	.889	0.046

Columns indicate absolute mean pre- and posttest values, percent change (Δ%), paired t-test significance (*p*), and the effect size (cohens *d*) (normalized to body mass for strength). Nm, newton meter; aROMmax, maximum active range of motion; pROMsub, submaximal passive range of motion; pROMmax, maximum passive range of motion; °, angle degree; °/s, degrees per second (movement velocity); mm, millimeter; mm^3^, cubic millimeter; values in brackets, standard deviation.

Muscle structural changes were calculated for the supraspinatus muscle and showed a non-significant result (Wilks-Lambda = .363; F(2,8) = 7.03; *p* = .017; ηp^2^ = .637). However, post-hoc tests for the pre-post differences can be found for FL (t(9) = 3.53; *p* = .003; d = 1.117) and FV (t(9) = 3.82; *p* = .002; d = 1.207). The Infraspinatus muscle did not show statistically significant pre-post differences in multivariate tests (Wilks Lambda = .157; F(2,5) = 2.150; *p* = .347; ηp^2^ = .843) and post-hoc t-tests (Table 2). Furthermore, no changes were found for the metric FA.

### Internal rotator cuff muscles functional changes

A three-factors repeated measures MANOVA were calculated for the internal rotator cuff muscles strength, showing no significant changes in all metrics. Furthermore, a one-way MANOVA for the stretching tests showed no significant changes (Table 3).

**Table 3:** Post-hoc comparisons for the internal rotator cuff muscles.

Muscle	parameter		Pre	Post	Δ%	*p*	*d*
Internal rotator cuff muscles	Strength eccentric (Nm)						
	Mean torque	30°/s	31.35 (9.45)	33.02 (10.41)	+5.33	.363	0.089
		60°/s	39.54 (8.39)	40.54 (10.83)	+2.53	.432	0.044
		180°/s	41.64 (9.85)	41.86 (13.65)	+0.55	.475	0.016
	Peak torque	30°/s	45.71 (15.14)	52.42 (16.85)	+14.68	.139	0.282
		60°/s	51.39 (13.60)	55.20 (14.22)	+7.42	.258	0.166
		180°/s	53.59 (17.00)	55.58 (19.29)	+3.71	.429	0.046
	Strength concentric (Nm)						
	Mean torque	30°/s	22.63 (6.14)	21.90 (6.74)	-3.22	.337	0.107
		60°/s	27.33 (5.54)	26.67 (7.19)	-2.40	.354	0.095
		180°/s	24.56 (5.91)	24.65 (6.58)	+0.36	.474	0.017
	Peak torque	30°/s	33.60 (9.39)	36.11 (10.30)	+7.45	.295	0.138
		60°/s	35.98 (8.45)	37.80 (10.31)	+5.06	.355	0.095
		180°/s	31.70 (8.69)	32.37 (8.48)	+2.13	.459	0.026
	Flexibility						
	Active stretch	aROMmax (°)	98.93 (0.88)	101.53 (7.55)	-2.63	.092	0.349
	Passive stretch	pROMsub (°)	82.88 (7.84)	81.24 (8.20)	-1.98	.142	0.278
		pROMmax (°)	109.44 (7.34)	108.33 (8.56)	-1.01	.226	0.193

Columns indicate absolute mean pre- and posttest values, percent change (Δ%),paired t-test significance (*p*), and the effect size (cohens *d*) (normalized to body mass for strength). Nm, newton meter; aROMmax, maximum active range of motion; pROMsub, submaximal passive range of motion; pROMmax, maximum passive range of motion; °, angle degree; °/s, degrees per second (movement velocity); values in brackets, standard deviation.

### Changes in eccentric and concentric torque-angle curves

**Fig 4:**
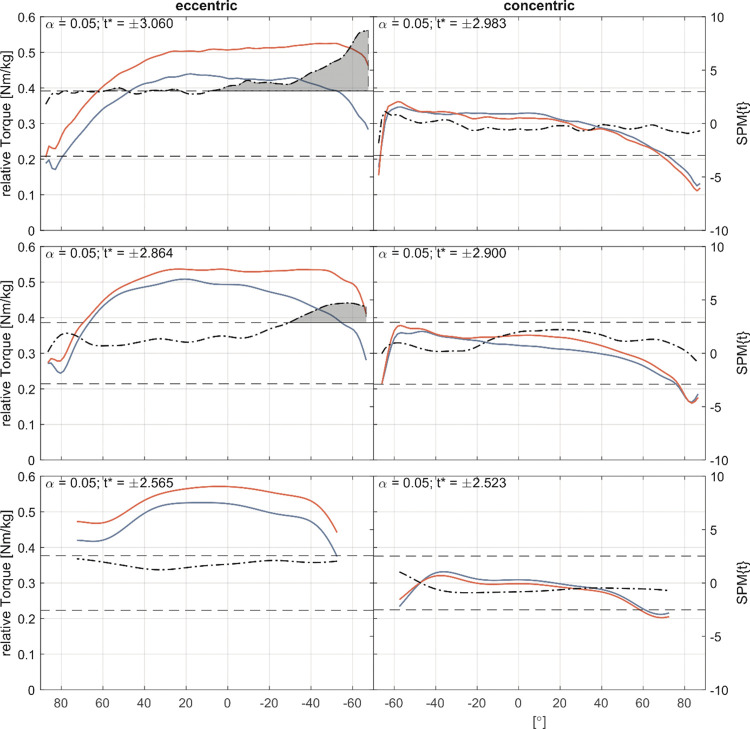
External rotator cuff muscles torque-angle-curves. Pre- (blue) and Post-measurement (red) torque-angle curves for 30°/s, 60°/s and 180°/s (top-to-bottom) for the eccentric (left column) and concentric (right column) strength. The dash-dotted line indicates the *t*-values calculated by the Statistical Parametric Mapping method (SPM1d) for each angle, the dashed line indicates the critical *t*-value. If the *t*-values exceed the critical value, significant differences exist between pre- and post-measurement and are marked as grey area.

**Fig 5:**
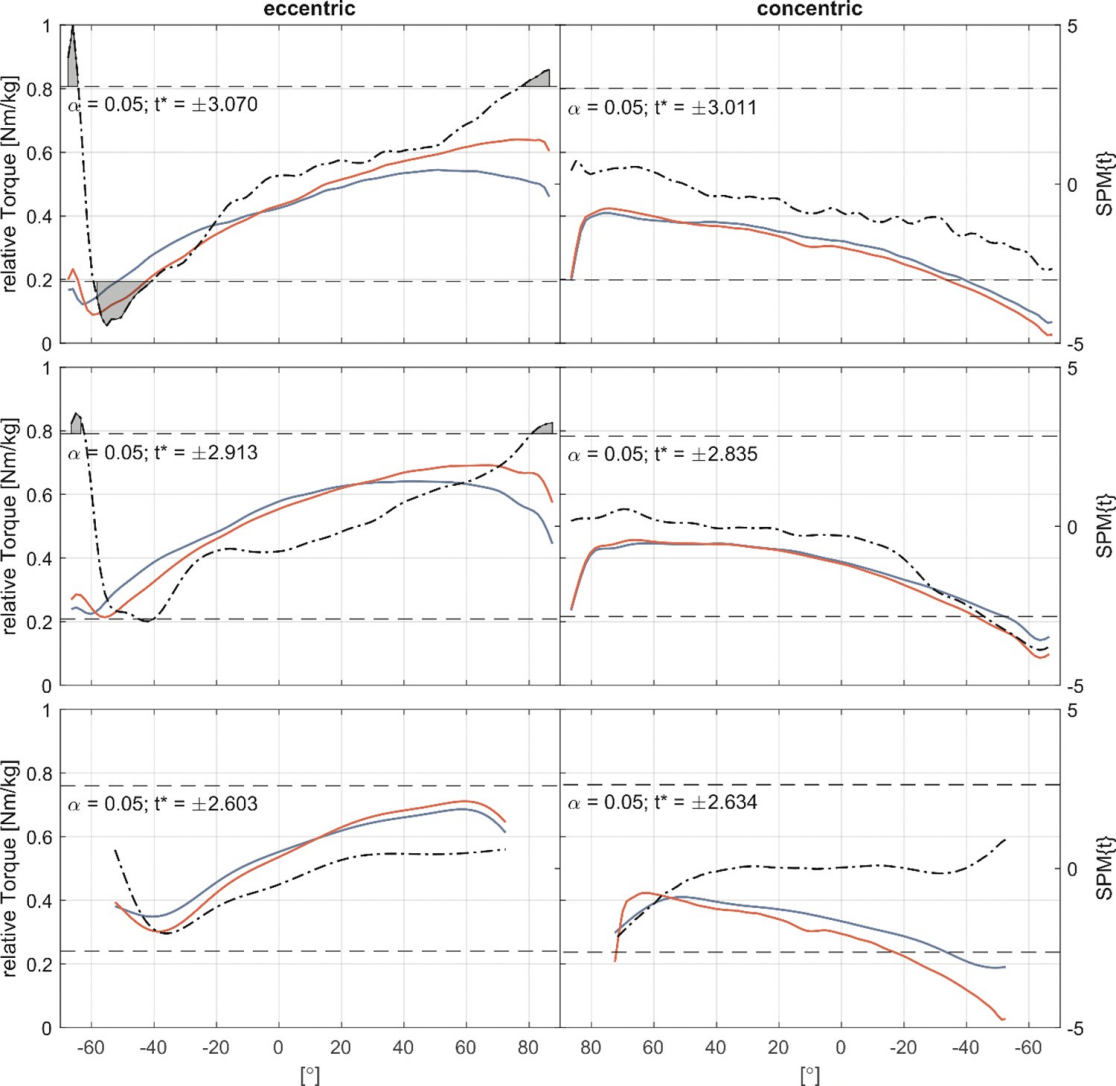
Internal rotator cuff muscles torque-angle-curves. Pre- (blue) and Post-measurement (red) torque-angle curves for 30°/s, 60°/s and 180°/s (top-to-bottom) for the eccentric (left column) and concentric (right column) strength. The dash-dotted line indicates the *t*-values calculated by the Statistical Parametric Mapping method (SPM1d) for each angle, the dashed line indicates the critical *t*-value. If the *t*-values exceed the critical value, significant differences exist between pre- and post-measurement and are marked as grey area.

For the external rotator cuff muscles strength tests, SPM1d analysis of the torque-angle relationship revealed significant changes between neutral position and 60° internal rotation in the eccentric test with an isokinetic speed of 30°/s. The eccentric test with 60°/s movement velocity showed a significant increase in torque between 30 and 60° internal rotation (Fig 4). SPM1d analysis of the internal rotator cuff muscles showed a significant change in curve morphology in the internal rotation in the 60–40° ROM and in the final ROM above 80° (Fig 5).

### Changes in passive torque-angle curves

**Fig 6:**
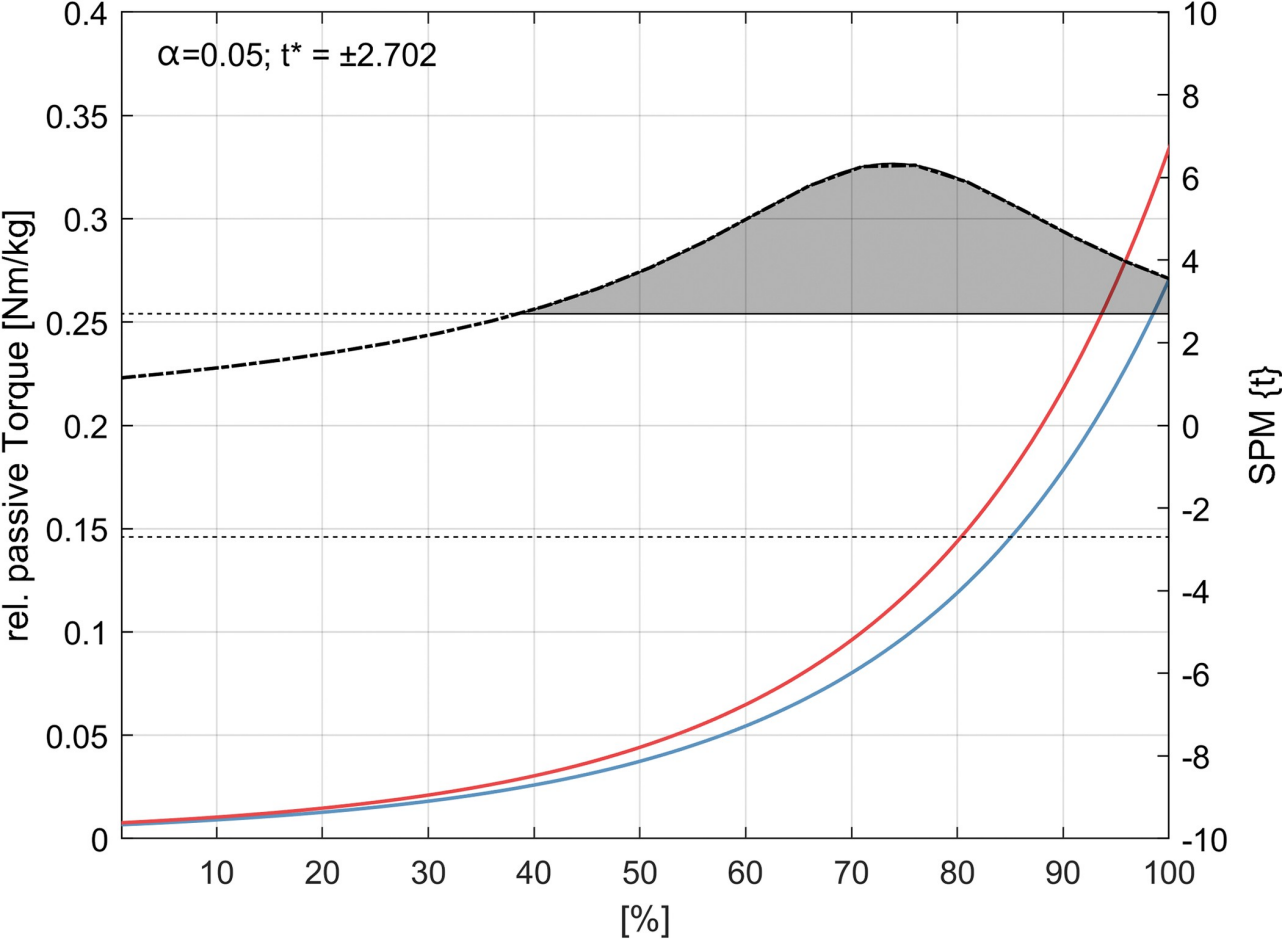
External rotator cuff muscles passive stretching torque-angle-curves. Pre and post group torque-angle curves (blue and red) extrapolated from the individual offset start angle of each subject to 100° of internal rotation (100% extension during testing). Newton meters (Nm) are expressed relative to the body mass (kg) of each subject. The dotted line indicates the *t*-values calculated by the statistical parametric mapping method (SPM1d) for each angle, the dashed line indicates the critical *t*-value. Significant differences are indicated by the gray area in the figure.

**Fig 7:**
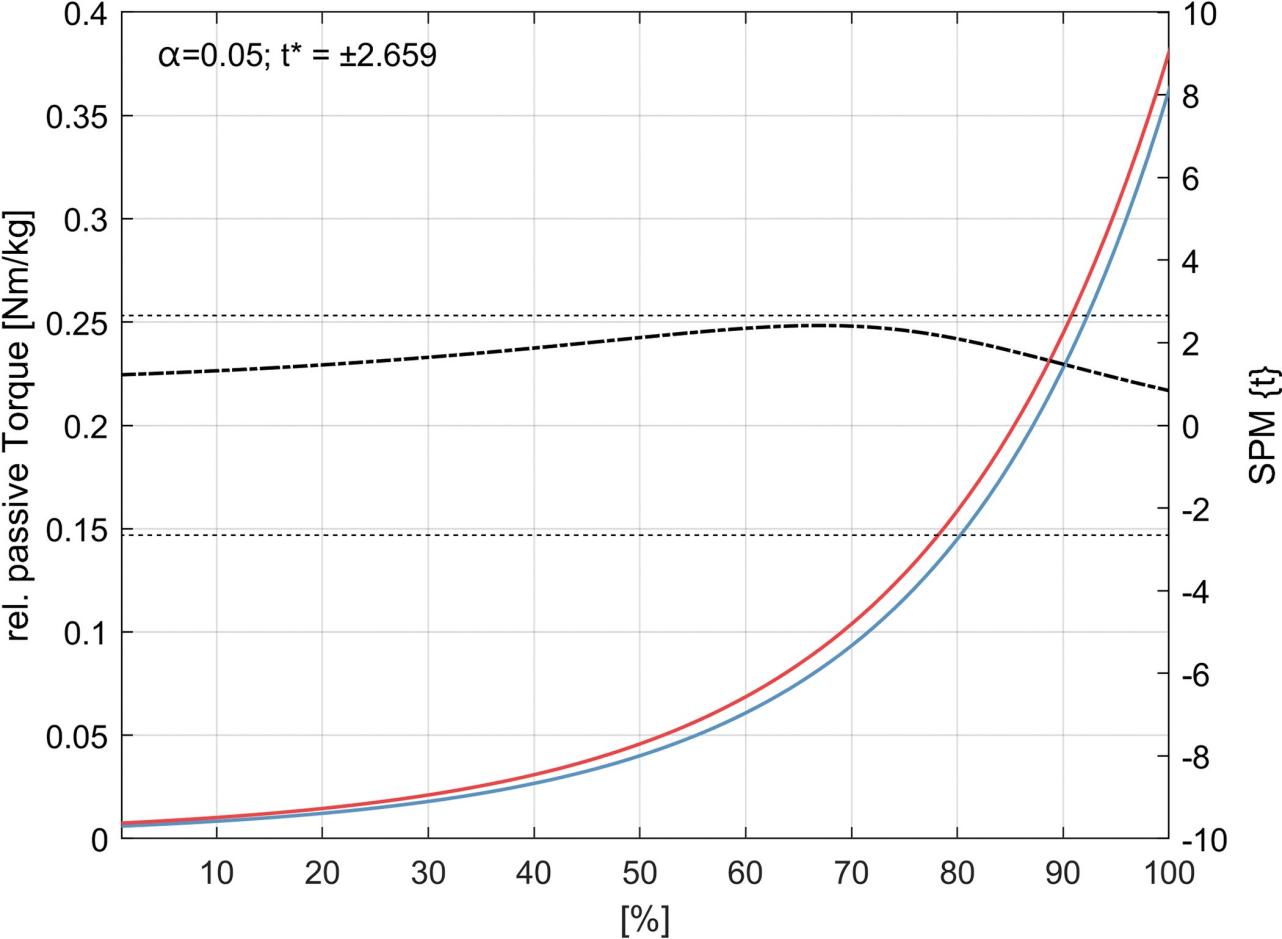
Internal rotator cuff muscles passive stretching torque-angle-curves. Pre-post (blue-red) group torque-angle curves extrapolated from each subject’s individual offset start angle to 130° of external rotation (100% extension in test). Newton meters (Nm) are relative to each subject’s body mass (kg). The dash-dotted line indicates the *t*-values calculated by the Statistical Parametric Mapping method (SPM1d) for each angle, the dashed line indicates the critical *t*-value. Significant differences are indicated by the gray area.

For the external rotator cuff muscles passive stretching tests SPM1d analysis showed an increase in passive stretching torque-angle relationship from 40° ROM to 100° ROM (Fig 6). No differences in passive torque-angle relationship were statistically confirmed for the internal rotator cuff muscles (Fig 7 and Table 3).

## Discussion

The purpose of this study was to determine if eccentric resistance training can produce similar and plausible results for the shoulder compared to other lower extremity studies and to evaluate the study methodology. We hypothesized that six weeks of 30°/s isokinetic eccentric resistance training targeting the external rotator cuff muscles, would have positive effects on eccentric strength, active and passive stretching behavior, and supraspinatus and infraspinatus muscles FA, FL and FV. However, the absolute values of eccentric strength increased significantly by +24% in the 30°/s test in particular in the final ROM of the isokinetic phase (Fig 4). Flexibility tests showed a significant decrease in both the aROMmax (-7%) and the pROMmax (-4%), while the passive torque-angle relation increased. In addition, the supraspinatus FL increased significantly by +16% and the FV increased by +19%. Other primary outcome measures, such as concentric strength for the trained external rotator cuff muscles, changed up to +7% but not statistically significant. Secondary outcome measures showed no statistically significant changes in the internal rotator cuff strength and flexibility tests.

Strength tests results are plausible and comparable to other lower extremity eccentric-only training studies, explaining a 19% gain in eccentric strength and a 9% improvement for the concentric strength [28]. Several mechanisms and adaptations may account for the changes in strength. First, the changes in strength and FV are addressed in terms of calculated fiber elongation [24] and the documented right shift in the torque-angle relationship (Fig 4), which has been discussed as a functional correlate of sarcomere formation [24, 40, 48]. Second, the changes found are primarily concerned with possible changes in FV as a possible surrogate parameter for physiological cross-sectional area of a muscle.

As multiple tissues define the ROM of the shoulder, a decrease in flexibility can be attributed to the function and structure of the glenohumeral joint. Since the shoulder joint consists of at least three other joints, not only the stabilizing muscles, but also passive structures such as the capsule determine its function. Therefore, Zandt et al. [49] expected low adaptability for rotator cuff muscles following eccentric resistance training. Furthermore, Camargo and colleagues [29], summarized that the stimulation of the target muscle seems to be problematic for shoulder rotational movements. However, this study showed interesting changes in joints flexibility while parametrizing the passive stretching torque-angle curves and extracting parameters that characterize the changes in the stretching behavior until the final stretch was reached. Our results show that a decrease in ROM after eccentric resistance training can be explained by an increase in the torque-angle relationship during the passive stretch test. This can be explained by changes in stiffness either due to addition in sarcomeres in series or due to chronic titin elongation and sarcomere lengthening [50, 51]. Therefore, this approach provides new information on the stretch behavior of human shoulder external rotator cuff muscles after resistance training without using imaging techniques to quantify tissue stiffness [52].

This study showed a 16% increase in FL following eccentric training. Changes in FL can be attributed to either sarcomere elongation or sarcomerogenesis [50, 51]. As a functional indicative for sarcomerogenesis [24, 40, 48], the present study found a positive change in FL and in torque-angle relationship. Interestingly, the found increase in FL is in contrast to a similar study by Kim and colleagues [34]. They also performed eight weeks of intense eccentric isokinetic training for the supraspinatus muscle but with an abduction exercise; and reported no changes for the fiber bundle length accessed with ultrasound [34]. However, Kim and colleagues [34] also found a positive functional and structural difference compared to a concentric training group.

Compared to other eccentric training studies, this change in FL shows to be 6% above the mean fiber lengthening reported for the lower limbs in a systematic review [28]. However, this review article also showed a wide range for adaptation, ranging from a 3% non-significant decrease in FL [53] to a possible 33% increase in FL for the hamstring [54]. Since the review article showed small differences in training method and exercise load between these two studies, the ultrasound imaging method applied was discussed as a possible reason for measuring different values in FL changes. Compared to MRI methods, 2D ultrasound imaging appears to be less objective and reliable [55]. To our knowledge, Suskens and colleagues [37] published the first study using MRI-based mDTI for this purpose and observed a 14% (2 cm) increase in the semitendinosus muscle FL after Nordic hamstrings training. Interestingly, mDTI did not reveal any FL changes in the biceps femoris long head, which is in contrast to most ultrasound studies in this research area [20, 28]. However, while Suskens and colleagues [37] trained the lower extremities, the present study appears to be the first eccentric training shoulder study using mDTI. In terms of baseline FL and FV, the demonstrated results appear to be comparable to anatomical studies indicating a wide range of mean fiber length between 2.8 and 8.3 cm [56, 57] and 6.6 cm for the infraspinatus muscle [57]. Since the population, measurement method, and number of fascicles measured can lead to incomparability between studies [56], the present study appears to show results within the range for typical structural shape and adaption capacity for the target muscles.

### Limitations

There are several limitations that should be noted. First, this is a preliminary study without a control group and resulting in some invalid data for analysis. However, this study was conducted to test the experimental design and to show the potential results for physically active men to provide a basis for future studies. Second, the sample size limited the statistical power and therefore some variables showed some trends but did not reach significance regardless of the analysis methods performed. Therefore, analysis revealed low sample size for active stretching tests and MRI and these results have to be interpreted with caution. However, to the best of our knowledge only Kim and colleagues [34] conducted a study comparable to the present study while using ultrasound imaging and did not report effect sizes or sample size calculations for the FL. Although the supraspinatus muscle is not comparable to the well-studied lower limb muscles, ultrasound imaging studies [39, 58, 59] or DTI studies [37] suggested group-sizes of at least 16 participants. Therefore, the sample size of the present study is a limitation. Third, the eccentric external rotation exercises used in this study may not account for strength training-induced changes in muscle architecture to the same extent as abduction exercises [29, 30, 34]. However, because of the widespread deficit in internal rotation of the glenohumeral joints as a risk factor for injury [60, 61], in the present study an eccentric rotational exercise has been used to directly target the major functional limitations of most subjects in overhead sports. Furthermore, the training with an isokinetic machine was very specific, whereas several subjects showed irritations in the torque-angle curves during training and testing. Therefore, it is possible that free-weight eccentric resistance training may produce at least the same effects as isokinetic training in such a population. Fourth, the stretching tests may not be comparable to other studies that primarily used physical therapists [29, 30, 34] to determine ROM of the glenohumeral joint. However, as most studies determine submaximal ROM without reaching an individual maximum stretch pain and with low standardization in how ROMmax is defined [21], this test is able to show stretching behavior in a standardized manner until a predefined torque or angle of motion is reached. Last, differences in anatomical and diffusion-weighted MRI scan parameter settings led to difficulties in image registration and resampling. Because of these difficulties, four subjects were excluded from the infraspinatus analysis due to a proximally restricted field of view. In addition, the stopping criteria for fiber tracking resulted in interpolations and different tract densities between subjects which was also explained in a previous reliability study [47]. Based on this issue, another limitation of the present study is that it does not include further reliability and validity analysis. However, DTI represents the diffusion of tissue water molecules to calculate fiber length based on reconstructed tensors. Therefore, DTI results should be interpreted with caution.

### Conclusion

Following this study, it was interpreted that eccentric training induces improvements in functional and structural measures of the shoulder external rotator cuff muscles. To our best knowledge, this is the first study of eccentric shoulder training using mDTI and complex isokinetic muscle function diagnostics. Therefore, the strength of the present study is that it provides more insight into three-dimensional muscle adaptation after eccentric training. Another strength of the study is its multidirectional perspective. Even the innovative implementation of a new method to access passive stretching behavior using an e-function to extract several stretching parameters within a torque-angle curve showed interesting insights into muscle physiology. In terms of practical implications, eccentric training can be considered as a promising primary prevention strategy for the shoulder. However, it remains to be proven whether these effects are still significant when compared to conventional shoulder prevention strategies in a randomized-controlled trial. Furthermore, it is unknown whether this training program would produce similar results for a chronic high-impact shoulder in a sample of overhead athletes. In terms of research, the applied functional and structural diagnostics revealed to be time-consuming and complex in terms of data processing. Therefore, future studies should consider minimizing functional tests and using automated muscle segmentation for mDTI analysis.

## Supporting information

S1 Appendix Appendix_Passive stretching torque-angle curve extrapolation a validity analysis. (DOCX)
