# Supplementary material for: Effect of isokinetic eccentric training on the human shoulder strength, flexibility, and muscle architecture in physically active men: A preliminary study
Source: PLoS One. 2023 Dec 19;18(12):e0293439. doi: 10.1371/journal.pone.0293439 (PMC10729965; doi:10.1371/journal.pone.0293439)
Supplement: S1 Appendix — (DOCX) [file pone.0293439.s001.docx]

**Appendix: Passive stretching torque-angle curve extrapolation: a validity analysis that compares extrapolated curves to actual measurements**

**Aim.** The purpose was to assess the flexibility of the shoulder internal rotators within a wide ROM (130° external rotation) by extrapolating recorded passive torque curves, while testing a narrower ROM (110°) to protect subjects inexperienced in overhead activities. The second objective was to investigate the reliability of passive torque determination at a specific external rotation angle (130°) with different data sets.

**Method.** 26 passive shoulder tests were performed on overhead athletes using an isokinetic dynamometer (IsoMed 2000, D.+R. Ferstl GmbH, Hemau, Germany). Subjects were tested in the supine position with 90° shoulder abduction and 90° elbow flexion. The ROM ranged from neutral to 130° of external rotation, so that only the internal rotators of the shoulders were stretched during the test. The dynamometer speed was set at 5°/s in external rotation and 10°/s in the opposite direction. Each test consisted of five repetitions with alternating directions. The last three repetitions were used for further data analysis. Once the data were recorded, they were truncated to 120° and 110°, resulting in three data sets.

The data were processed by fitting the torque curves T (*α*) with an e-function and calculating the passive torque at 130° for each trial:

$${T(\alpha)=s_{1}\cdot e}^{(s_{2}\cdot\alpha)}+s_{3}$$

The fit uses data points from the minimum torque, from which the torque begins to increase, until the last data point for each set (Fig 7).

After fitting, the parameter s3 was extracted in order to be independent of inaccuracies in the positioning of the subject in relation to the dynamometer, since the curvature of the passive torque envelope, determined by the parameters s1 and s2, is used to assess the flexibility of the shoulders. For each test, the median fit of the three repetitions was calculated and used to determine the torque at 130° external rotation for each subject and data set. A Pearson correlation was then performed between the data sets.


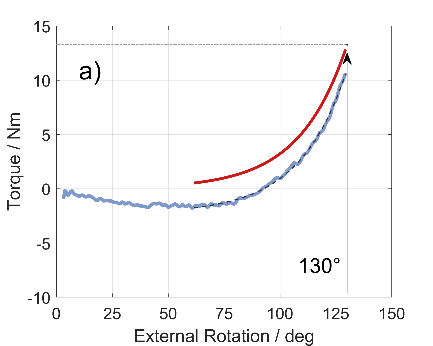

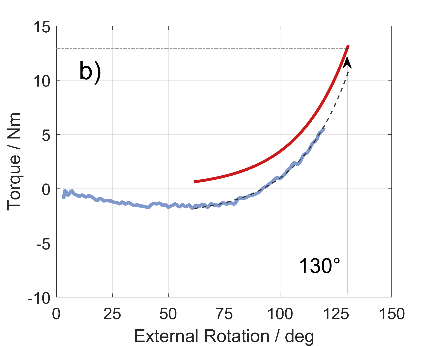

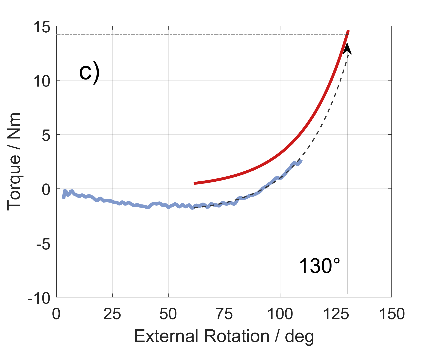


**Fig 7. Example plots for original curves (a), curves truncated to 120° (b) and 110° (c), respectively.** The recorded torque curves are in blue, the original fits are shown as black dashed lines, and the fits corrected by the offset of parameter s_3_ and further used to calculate the torque at 130° are the red lines. Note that only the ascending portion of the blue curves were used for curve fitting, starting at the angle of minimum torque.

**Results and Discussion.** The extrapolated curves showed excellent ICC (>.93) and correlation (r >.938) when compared to actual measured torque-angle curves (Table 4-5). Therefore, this method was interpreted as an alternative to actual measurements. As an advantage, the extrapolation method allows for more standardization of shoulder rotational flexibility measurements, as the measurement results may be largely based on the glenohumeral joint. Second, it reduces the risk of discomfort or pain during the extension test, as pain tolerance may be another important determinant of extension ability.

**Table 4. ICC.**

| **Comparisons** | **ICC** | **CI 95%** | | **F-Test** | | | |
| --- | --- | --- | --- | --- | --- | --- | --- |
|  |  | **<** | **>** | **F-value** | **df** | **df2** | ***p*** |
| 120-130° extrapolation vs. 130° actual measurement | .950^c^ | .863 | .979 | 24.667 | 25 | 25 | <.001 |
| 110-130° extrapolation vs. 130° actual measurement | .931 | .777 | .973 | 19.330 | 25 | 25 | <.001 |

ICC, Intra-class correlation coefficient; CI, confidence interval; df, degrees of freedom; *p*, significance.

**Table 5. Pearson correlation analysis.**

| **Comparisons** | ***r*** | ***p*** |
| --- | --- | --- |
| 120-130° extrapolation vs. 130° actual measurement | .949^c^ | <.001 |
| 110-130° extrapolation vs. 130° actual measurement | .938 | <.001 |

*r*, Pearson correlation coefficient; CI, confidence interval; df, degrees of freedom; *p*, significance.
